# Hype or hope of hyaluronic acid for osteoarthritis: Integrated clinical evidence synthesis with multi-organ transcriptomics

**DOI:** 10.1016/j.jot.2021.11.006

**Published:** 2022-01-15

**Authors:** Kun Zhao, Ya Wen, Varitsara Bunpetch, Junxin Lin, Yejun Hu, Xiaoan Zhang, Yuan Xie, Shufang Zhang, Ouyang Hongwei

**Affiliations:** a Dr. Li Dak Sum & Yip Yio Chin Center for Stem Cells and Regenerative Medicine, and Department of Orthopedic Surgery of the Second Affiliated Hospital, Zhejiang University School of Medicine, Hangzhou, China; b Department of Sports Medicine, Zhejiang University School of Medicine, Hangzhou, China; c Zhejiang University-University of Edinburgh Institute, Zhejiang University School of Medicine, and Key Laboratory of Tissue Engineering and Regenerative Medicine of Zhejiang Province, Zhejiang University School of Medicine, Hangzhou, China; d China Orthopedic Regenerative Medicine Group (CORMed), Hangzhou, China

**Keywords:** Hyaluronic acid, Osteoarthritis, Meta-analysis, Randomized controlled clinical trails, Multi-organ transcriptomics

## Abstract

**Background:**

Intra-articular injections of hyaluronic acid (HA), the United States Food and Drug Administration approved treatment and widely utilized to delay or reserve the progression of the osteoarthritis (OA) involves. However, this treatment has shown controversial results through various clinical practice guidelines and meta-analysis evaluations, warrants more advanced researches on its safety and effectiveness.

**Methods:**

A novel strategy of integrating medical informatics and bioinformatics was utilized. An updated meta-analysis of 16 randomized controlled trials (RCTs) out of 1820 articles was conducted, in combination with a high throughput body-wide-organ-transcriptomic (BOT) RNA-sequencing (RNA-seq) and *in vitro and vivo* experiments to evaluate the effect of HA at local and systemic levels, revealing the underlying mechanism.

**Results:**

A sensitivity analysis was performed restricting to high quality RCTs, no significant effect of HA treatment was found on pain relief and functional improvement. Descriptive analysis of RNA-seq using Gene Ontology and Kyoto Encyclopedia of Genes and Genomes pathway analysis revealed biological process related to innate immune responses and apoptosis; BOT analysis revealed differential gene expressions (DEGs) in cartilage, lymph node, spleen, kidney, and liver, with immune cell proliferation in immune-related organs. *In vitro,* HA-coated plates were shown to induce macrophage responses; *in vivo* histological images revealed knee joint, liver, and kidney with damaged/abnormal morphologies, while immune cell proliferation was observed in the lymph node and spleen and it was found that there was no significant difference in the treatment effect for OA animal model.

**Conclusion:**

Conclusively, integration of meta-analysis with bioinformatics analysis exhibited that HA induces inflammatory responses both locally and systematically and not benefit for OA treatment, thus limiting the regeneration and leading to some organ-specific pathogenesis. The strategy and findings will be of important for guiding future long-term clinical studies.

**The Translational potential of this article:**

This study illustrated that the administered HA activated both systemic and local pro-inflammatory immune responses, possibly limiting its efficacy. This novel unique strategy proposed in this study can be utilized and adapted for future meta-analysis and bioinformatics study.

## 1 Introduction

Although knee osteoarthritis (OA) is a common chronic degenerative joint disease in older adults, OA is recognized to be triggered by more than the age-associated “wear-and-tear” on the joints. Damages to a joint—whether through physical activities or traumas, or even simple daily movements—can lead to joint degeneration, causing those affected to persistently suffer from pain, stiffness, and limited mobility.

Over the last decades, intra-articular injections of hyaluronic acid (HA) are widely utilized in the therapeutic management of knee OA since it received United States Food and Drug Administration approval in 2001. Even though most individual studies reported positive outcomes, there remains controversy regarding the clinical effectiveness of intra-articular injected HA, with conflicting conclusions of the collective data between studies and with divergent levels of recommendation by international and national societies. The Cochrane Collaboration and American College of Rheumatology reported that evidence was adequate to encourage the administration of HA for knee OA treatment, the 2013 American Association of Orthopedic Surgeons and 2014 UK National Institute of Care Excellence guidelines stated otherwise [[1], [2], [3], [4]]. While the Osteoarthritis Research Society International guidelines were more favorable and reported intra-articular HA injections to be “uncertain” for knee OA [5]. These contradictory recommendations were formed on the basis of different meta-analyses. Numerous updated meta-analyses have been added to this research area since the publication of the guidelines. A 2015 meta-analysis found there was no clinically important benefit compared with placebo [6]. By contrast, a 2019 systematic review showed significant pain reduce for early-moderate knee OA [7].

Nevertheless, even though meta-analysis is a powerful way to deal qualitatively with varying study results, it is a non-experimental study solely providing statistically calculated numbers. Most meta-analysis related studies focus on answering a simple *yes* or *no* question, instead of *how* or *why*; moreover, very limited available data investigated the effect of HA on the whole body.

Significant advancement in high-throughput sequencing technology has transformed almost every corner of biomedical sciences, with the ability to detect even the slightest alteration in cells and tissues as well as the genomic level. In this study, a novel strategy of integrating meta-analysis with multi-organ transcriptomics was utilized to evaluate the safety and effectiveness of HA both locally and systemically. In addition to an updated meta-analysis, RNA-sequencing was conducted to systematically study the body-wide-organ transcriptomic response and the underlying specific biochemical interactions in mice to the infiltrated HA. The impact of HA was further investigated in both *in vitro* cell culture and *in vivo* animal-based model to evaluate its efficiency and safety.

## 2 Method

### 2.1 Meta-analysis comparing HA and saline

A comprehensive search of the following electronic databases was conducted: PubMed, the Cochrane Central Register of Controlled Trials(CENTRAL), EMBASE, the specific search strategy is shown in Table S1. The search ended on January, 2021. The inclusion criteria were listed as follows: 1) Randomized controlled trials (RCTs) comparing HA and saline treatments on adult patients (aged over 18 years old) with knee OA and published trials available as full articles in English; 2) key outcome indicators such the Western Ontario McMaster Universities Arthritis Index (WOMAC), visual analog scale (VAS), Lequense Index, or adverse events were utilized to analyzed the data. Duplicate publications and conferences were not included in the final selection. Two reviewers independently reviewed and collected relevant data from the selected reports. When a report indicated more than one pain or function outcome measure, we gave preference to the WOMAC pain and function measures as it is the most commonly used and thoroughly validated instrument for assessing patients with OA. The methodological quality for the included RCTs was independently evaluated by two reviewers based on Cochrane risk of bias criteria. The trials were graded as low, high, or moderate quality based on the criteria as described by Zhao et al. [8]. Review Manager (RevMan 5.3) was used to performed the analysis. For each trial, continuous outcomes for the meta-analysis were evaluated and reported as standardized mean differences (SMDs) with 95% confidence intervals, and dichotomous outcomes are illustrated as risk ratios (RRs) with 95% confidence intervals. Statistical heterogeneity was calculated using the I^2^ test, a random-effects model was applied to pool the data. Sensitivity analysis was performed by restricting the analyses to high quality RCTs. If multiple treatment arms were reported in a single trial, only the relevant arm was included in the analysis. The significance of the pooled effects was accessed by a Z test, and a *P* value of less than 0.05 was considered significant.

### 2.2 Isolation and culture of primary mouse chondrocytes

All procedures for the animal study were approved by the ∗∗∗∗∗∗). Primary mouse chondrocytes were extracted from the femoral condyles and tibial plateaus of postnatal day 0–1 C57B1/6 mice [9]. Chondrocytes were maintained as a monolayer in DMEM/F-12 supplemented with 10% FBS and 1% P/S at 37 ​°C; chondrocytes between 1st to the 3rd passage were used for experiments.

To extract primary mouse macrophages, 2 ​ml thioglycollate solution (4%) (Merck) was injected into the peritoneal cavity; the solution was then collected after 4 days to isolate macrophages then seeded onto the HA-coated or control plates. After 6 and 12 ​h, culture supernatants were analyzed by ELISA for IL-1β and TNFα.

### 2.3 RNA-seq and data analysis

For the different groups, RNAs were isolated, sequenced, and analyzed from four duplicates. RNA-seq procedures were modified from a previously published method [10]. Trizol reagent (TAKARA), SuperScript II reverse transcriptase (Invitrogen), and NEBNext mRNA second strand synthesis kit (NEB) was used for RNA extraction, reverse transcription, and double strand DNA synthesis, respectively. DNA was then washed with AMPure XP beads (Beckman Coulter); subsequently, sequencing library was produced using Nextera XT kit (Illumina) and sequenced on Illumina X-Ten platform. Bowtie2 with default parameters were applied to diagram sequence reads to reference genome mm10, and HTSeq were utilized to calculate per gene counts. DESeq2 R package was applied to determine differentially expressed genes (DEGs). DEGs were illustrated as fold-change ≥ 2 and p-value ​≤ ​0.05. Gene ontology (GO) and Kyoto Encyclopedia of Genes and Genomes (KEGG) pathways analysis was performed using DAVID online tool (http://david.ncifcrf.gov).

### 2.4 Preparation of HA-coated culture plates

The HA coating procedure was modified from a previously published method [11]. Briefly, purified HA (ACROS Organics™) was dissolved in Dulbecco's PBS at a concentration of 5 ​mg/ml, and then diluted into the desired concentration. Tissue culture plates (24 well plates) were then coated with 1 ​ml HA solutions overnight at 4 ​°C; uncoated wells were used as controls. Before cell seeding, wells were aspirated and washed with PBS twice to remove any aggregates and used immediately.

### 2.5 Cell proliferation assay

Chondrocyte proliferation was assessed using the Cell Counting KIT-8 (CCK-8) (Dojindo Molecular Technologies, Inc). Cells cultured on either non-coated control or HA-coated plates were first incubated in 10% CCK-8 solutions in a 5% CO_2_ incubator at 37 ​°C for 2 ​h; the media were then collected and the absorbance was measured at 450 ​nm. Cells were cultured in these two different coated conditions for 1, 3, and 5 days before evaluation.

### 2.6 Gene expression analysis by qPCR

The mRNA transcript levels of chondrogenic-related genes (*Acan, Col2a1,* and *Sox9*), as well as the inflammation related gene (*Mmp13*) within mouse chondrocytes cultured on HA-coated plates verses control were assessed by real-time PCR. At day 3, cells were harvested and lysed in Trizol (Invitrogen Inc., Carlsbad, CA, USA); mRNA was isolated according to the manufacturer's method. ReverTra Ace qPCR Master Mix kit (TOYOBO, Japan) and SYBR Green QPCR Master Mix (Takara) were used to perform reverse transcription and PCR, respectively, with a Light Cycler apparatus (Bio-rad, CFX-Touch). The relative expression level of target genes was then computed using the 2−ΔΔCt method. Each qPCR was performed on at least 3 distinct samples and results were illustrated as target gene expression normalized to the reference gene GAPDH.

### 2.7 In-vivo animal studies

Eight-week-old C57BL/6 mice (Zhejiang University) were utilized in this study. Saline control (n ​= ​10 joints) and HA solution (n ​= ​10 joints) (10ul) were injected into the intra-articular space in mice's knee joints under anesthesia (0.8% pentobarbital sodium). At 1-week post-injection, mice were sacrificed and four knee joints from to two groups were collected for RNA-sequencing, and six knee joints were histologically assessed. For knee joints, the images were blindly investigated by four investigators relying on the International Cartilage Repair Society (ICRS) macroscopic assessment scale for cartilage repair [12]. For lymph node and spleen, Image J was used to quantify the relevant area.

### 2.8 Animals and surgical induction of OA

Twelve-week old C57B1/6 mice (n ​= ​18) were obtained for DMM surgery. All surgery was connected under anesthesia with 0.8% pentobarbital sodium, and all surgeries were performed to minimize suffering. Saline control (n ​= ​9) and HA solution (n ​= ​9 joints) (10ul) were injected once a week into the intra-articular space in mice's knee joints under anesthesia. Mice were then sacrificed and collected knee joints at 1 week, 4 weeks and 6 weeks for histological analysis. The images were blindly investigated by four investigators relying on the International Cartilage Repair Society (ICRS) macroscopic assessment scale.

### 2.9 Statistical analysis

All outcomes are represented as mean ​± ​standard deviation (SD); differences between values were calculated using Student's t-test and ANOVA. The level of significance is illustrated as ∗ (*p* ​< ​0.05), and ∗∗(*p* ​< ​0.01); *p* ​< ​0.05 is considered to be statistically significant.

## 3 Results

### 3.1 Meta-analysis comparing HA vs saline injection in patients with OA

**Fig. 1:**
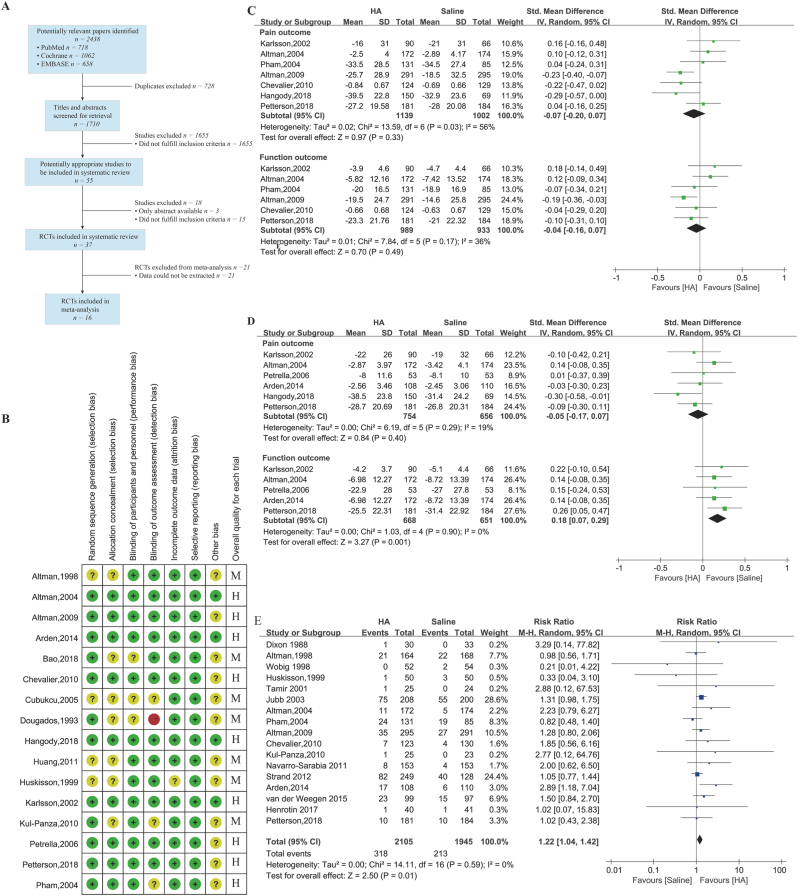
**Meta-analysis of intra-articular injected HA** (A) literature search and screening process (B) risk of bias of included trials (C). Sensitivity analysis of short-term result for HA compared to saline (D) Sensitivity analysis of long-term result for HA compared to saline (E). Adverse events results for HA compared to saline.

Fig. 1A shows the flow diagram of study selection. A total of 1820 studies were identified following the initial computerized search. Among these, 1769 records did not meet our inclusion criteria following a thorough review of the abstracts. The remaining 51 articles were retrieved for full text review; of these: follow-up less than 6 weeks (n ​= ​4), conference abstract (n ​= ​14) and data could not be extracted (n ​= ​17). Ultimately, the remaining 16 studies were evaluated and included in the meta-analysis [[13], [14], [15], [16], [17], [18], [19], [20], [21], [22], [23], [24], [25], [26], [27], [28]].

Table 1 illustrated the characteristics of the included records. The sample sizes in different studies varied from 40 to 586, with a total of 3221 patients; the HA group consisted of 1685 individuals whereas 1536 individuals were included in the control group. Fig. 1B illustrates the assessment of the risk of bias; the overall quality of the reported trials was acceptable, with 9 trials of high quality RCTs and no low quality trials.

**Table 1:** Characterize of included studies.

Trial	No. of Patients		Intervention		Outcome	Measurement timepoint from baseline
	HA	Saline	HA Type	Average Molecular Weight, kDa		
Dougados,1993	55	55	Hyalgan	500–730	Pain: VAS Function: Lequesne's function index Adverse events	7w, 52w
Altman,1998	105	115	Hyalgan	500–730	Pain: VAS Adverse events	9w, 12w, 16w, 21w,26w
Huskisson,1999	50	50	Hyalgan	500–730	Pain: VASFunction: Lequesne's index Adverse events	2 ​m, 4 ​m, 6 ​m
Karlsson,2002	90	66	Artzal	500–1000	Pain: VAS Function: Lequesne's index Adverse events	20w, 26w
Altman,2004	172	174	Durolane	Unclear	Pain:WOMAC pain Function: WOMAC function Adverse events	6w, 13w, 26w
Pham,2004	131	85	NRD101	1900	Pain: VAS Function: Lequesne's index Adverse events	52w
Cubukcu,2005	30	10	Hylan G-F 20	6000	Pain: VAS, WOMAC pain Function: WOMAC function	8w
Petrella,2006	53	53	Unclear	Unclear	Pain: VAS, WOMAC pain Function: WOMAC function Adverse events	6w
Altman,2009	291	295	Euflexxa	2400–3600	Pain: VAS Function: WOMAC function Adverse events	26w
Chevalier,2010	124	129	Hylan G-F 20	6000	Pain: WOMAC pain Function: WOMAC function Adverse events	26w
Kul-Panza,2010	25	23	Orthovisc	1500	Pain: VAS, WOMAC pain Function: WOMAC function, Lequesne's index Adverse events	14w
Huang,2011	100	98	Hyalgan	500–730	Pain: VAS, WOMAC pain Function: WOMAC function Adverse events	13, 25w
Arden,2014	108	110	Durolane	Unclear	Pain: WOMAC pain Function: WOMAC function Adverse events	6w
Bao,2018	20	20	ARTZ	620–1170	Pain: VAS, WOMAC pain Function: WOMAC function Adverse events	8w
Hangody,2018	150	69	Monovisc	Unclear	Pain: WOMAC pain Adverse events	6w, 12w, 18w, 26w
Petterson,2018	181	184	Monovisc	Unclear	Pain: VAS Function: WOMAC function Adverse events	8w, 12w, 20w, 26w

For our meta-analyses, we grouped the outcomes into two time points of measurement: short-term (<25 weeks), long-term (≥25 weeks). If studies reported multiple time points, we choose the one closest to 25 weeks respectively. Compare with saline control, HA provided significantly irrelevant short-term pain relief (SMD ​= ​−0.27, 95% CI: −0.45 to −0.10, *P* ​= ​0.002) and functional improvements (SMD, −0.38, 95% CI: −0.93 to −0.03, *P* ​= ​0.04), but the results were heterogeneous (I [2] ​= ​73%, 90%, respectively) (Figure S1A). HA also provided irrelevant long-term improvements in pain with high heterogeneity (SMD ​= ​−0.15, 95% CI: −0.28 to −0.03, *P* ​= ​0.02, I^2^ ​= ​62%) (Figure S1B). By performing a sensitivity analysis restricting to high quality RCTs, no significant effect of HA treatment was found on pain relief (SMD ​= ​−0.07, 95% CI: −0.20 to 0.07, *P* ​= ​0.33, I^2^ ​= ​56%) and functional improvement (SMD ​= ​−0.04, 95% CI: −0.16 to 0.07, *P* ​= ​0.49, I^2^ ​= ​36%) at short-term with satisfactory statistical synthesis (Fig. 1C). HA also provided no significant long-term improvements in pain (SMD ​= ​−0.05, 95% CI: −0.17 to −0.07, *P* ​= ​0.40, I^2^ ​= ​19%) and function (SMD ​= ​0.18, 95% CI: 0.07 to 0.29, *P* ​= ​0.001, I^2^ ​= ​0%) (Fig. 1D). Additionally, we synthesized inflammation-related adverse events outcomes related to knee pain, swelling, effusion, arthralgia by viewing the RCTs included in the systematic review (n ​= ​33); seventeen records were included to analysis [14,15,17,18,[21], [22], [23],^25^,[28], [29], [30], [31], [32], [33], [34], [35], [36]]. The pooling effects found a significant statistical difference (RR ​= ​1.22, 95% CI: 1.04 to 1.42, *P* ​= ​0.01, I^2^ ​= ​0%) (Fig. 1E), which means HA may be associated a greater risk of adverse events related inflammation reactions.

### 3.2 Local responses to HA

#### 3.2.1 Transcriptomic profiles of the influence of HA *in vitro*

**Fig. 2:**
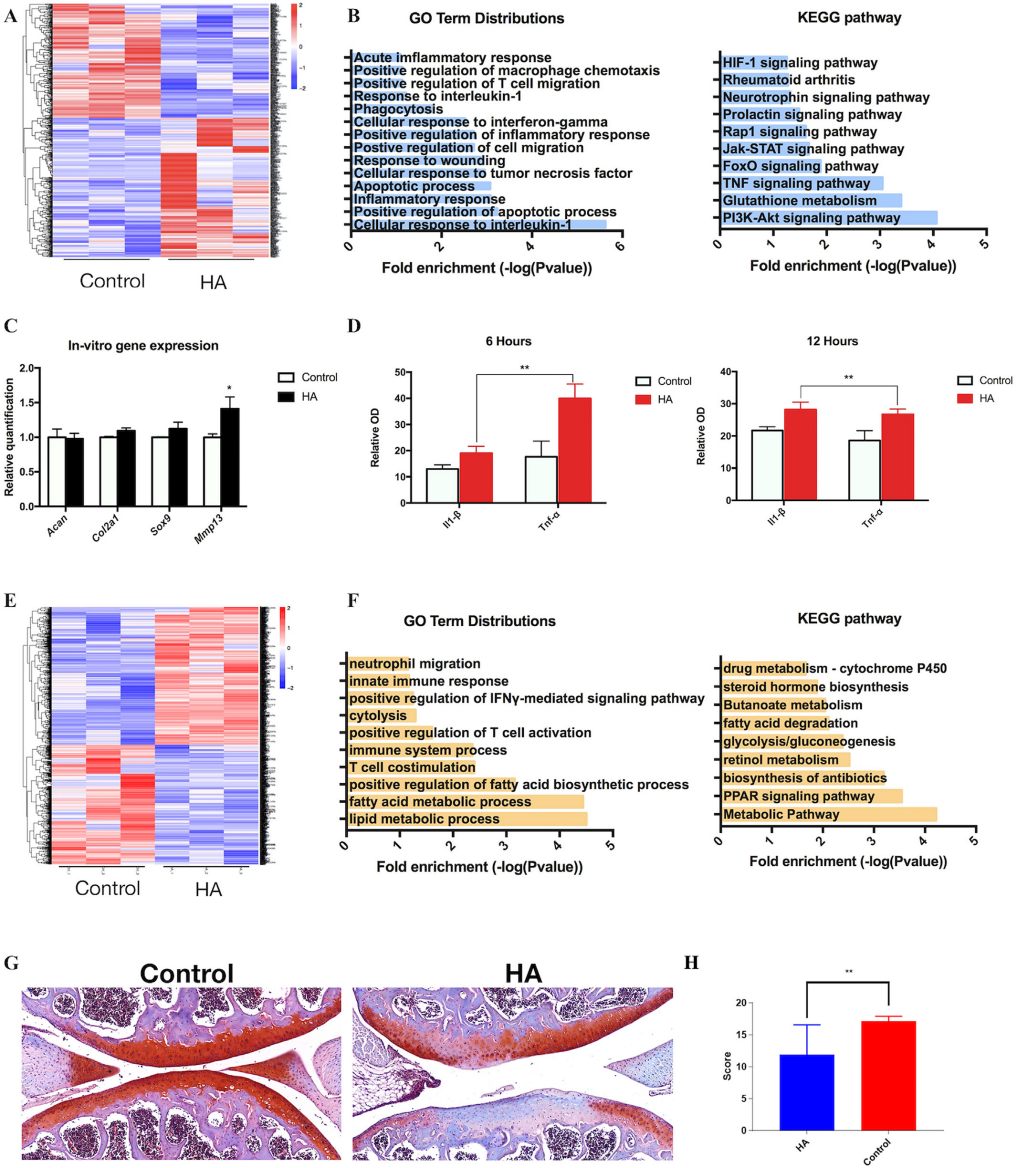
**The effects of HA on chondrocytes *in vitro* and knee joints *in vivo*** (A) A heatmap of differentially expressed mRNA levels from RNA-seq analysis performed on chondrocytes cultured on HA-coated plates (B) Gene Ontology (GO) enrichment and KEGG analysis of the upregulated genes in chondrocytes (C) Chondrocyte- and inflammation-related genes (*Col2a1, Acan, Sox9, Mmp13)*, and the (D) ELISA analysis of IL-1β and TNFα cytokine productions by macrophages cultured on HA-coated dishes (E) A heatmap of DEGs from RNA-seq performed on intra-articular HA-injected knee joints, as well as the (F) GO and KEGG analysis (G) Histological sections of the HA-injected knee joints at 1-week post-operation. Original magnification x20; scale bar: 50um (H) ICRS scoring on cartilage.

A whole transcriptome RNA sequencing was performed on chondrocytes cultured on HA-coated plates. CCK-8 analysis revealed that chondrocytes were able to effectively proliferation when cultured on HA-coated plates at a concentration of 0.5 ​mg/ml, consistent with another study (figure S2) [11]. DEGs induced in mouse chondrocytes in response to HA revealing significant changes in the transcriptomic profile when cultured on HA-coated plates (Fig. 2A, S3).

To gain further functional insights, the GO enrichment analysis was performed on chondrocytes. Results exhibited that GO terms cultured on HA-coated plates were predominantly related to acute inflammatory responses including response to oxidative stress, inflammatory response, positive regulation of apoptotic process, response to wounding, and positive regulation of inflammatory response (Fig. 2B), suggesting that HA induced reactive immune responses in chondrocytes. Furthermore, we also observed that GO terms related to cartilage and bone development such as chondrocyte development, cartilage development, and cartilage condensation were downregulated. Conclusively, these results indicated that HA, 1) induces inflammatory responses in chondrocytes and 2) negatively affects chondrocyte phenotype maintenance.

A KEGG pathway analysis of the upregulated genes group was also executed; the top pathways associated with the upregulated genes on chondrocytes cultured on HA are illustrated in Fig. 2B. Upregulated KEGG pathways include the Pl3K-Akt signaling pathway, glutathione metabolism, TNF signaling pathway, FoxO signaling pathway, as well as rheumatoid arthritis. Previous studies have illustrated that the P13K-Akt pathway was activated by inflammatory cytokines, leading to an increased production of MMPs and cartilage matrix loss [37,38]. Glutathione *metabolism* and TNF signaling pathway both play a crucial role part involving inflammation, including the metabolic integration during T cell responses [[39], [40], [41]]. From these results, it may be possible that immune responses to HA are activated via the glutathione and TNF signaling pathway, thus releasing inflammatory cytokines which then activated the P13K-Akt pathway.

#### 3.2.2 Effects of HA on primary mouse chondrocytes and macrophages

The impact of HA on chondrocytes’ phenotype maintenance was first evaluated by analyzing the expression of chondrogenic-related genes. Consistent with our meta-analysis results, the obtained data demonstrated that the expression of *Acan, Col2a1,* and *Sox9* in chondrocytes cultured on HA-coated plates was not significantly different when compared to the control group (Fig. 2C). However, chondrocytes cultured on HA-coated plates showed an upregulation of *Mmp13* when compared to the control. To further evaluate whether HA induces inflammatory responses, macrophages were also cultured on HA-coated versus non-coated plates where ELISA results revealed an upregulation of pro-inflammatory cytokines IL-1β and TNFα (Fig. 2D). Thus, these results revealed that HA did not enhance chondrogenic-phenotype maintenance, but induced inflammatory responses in chondrocytes *in vitro*.

#### 3.2.3 Impact of HA at the local injection site

HA was injected into healthy mouse knee joints; samples were then collected for whole-tissue RNA-sequencing and histological analysis. The control group was injected with PBS. Analysis of DEGs illustrated that HA injection significantly alters the transcriptomic profiles when compared to control samples (Fig. 2E). Consistent with the obtained *in vitro* results, GO enrichment analysis revealed terms related to lipid metabolic process, fatty acid metabolic process, T cell costimulation, immune system process, positive regulation of T cell activation, innate immune response, and positive regulation of interferon-gamma-mediated signaling pathway (Fig. 2F). Fatty acids were reported to be involved in inflammatory metabolic diseases such as inflammatory joint diseases, while interferon-gamma-mediated signaling pathway is involved in the regulation of the immune-derived cytokines in the innate and adaptive immune reactions [[42], [43], [44]].

Additionally, previous studies have noted that activation of the immune system demands a huge amount of energy; therefore, energy metabolism plays an essential part in the pathogenesis of diseases [45,46]. Consistently, our KEGG analysis revealed an upregulation of energy-related pathways such as the metabolic pathway and glycolysis/gluconeogenesis (Fig. 2F) in HA-injected samples. An upregulation of PPAR signaling pathway was also observed; a study has suggested that PPAR-γ might contribute to the persistent expression of pro-inflammatory cytokines in rheumatoid arthritis, and that they are responsible for the regulation of various genes involving in glucose homeostasis and lipid metabolism [47,48]. Thus, lipid and fatty acid processes may be upregulated through the PPAR signaling pathway, leading to pro-inflammatory responses in joints.

Moreover, in the HA-injected group, histological sections of the articular surface appeared to be irregular and unsmooth; there was also a reduction in the thickness of the articular cartilage (Fig. 2G). Representative images of the articular cartilage in HA-injected joints showed loss of Safranin-O/fast green staining with some structural alterations, illustrating a lower proteoglycan content. The mean ICRS score was approximately 1.4 times higher in the control group (Fig. 2H).

### 3.3 Transcriptomic profiling of multi-organ responses to HA

**Fig. 3:**
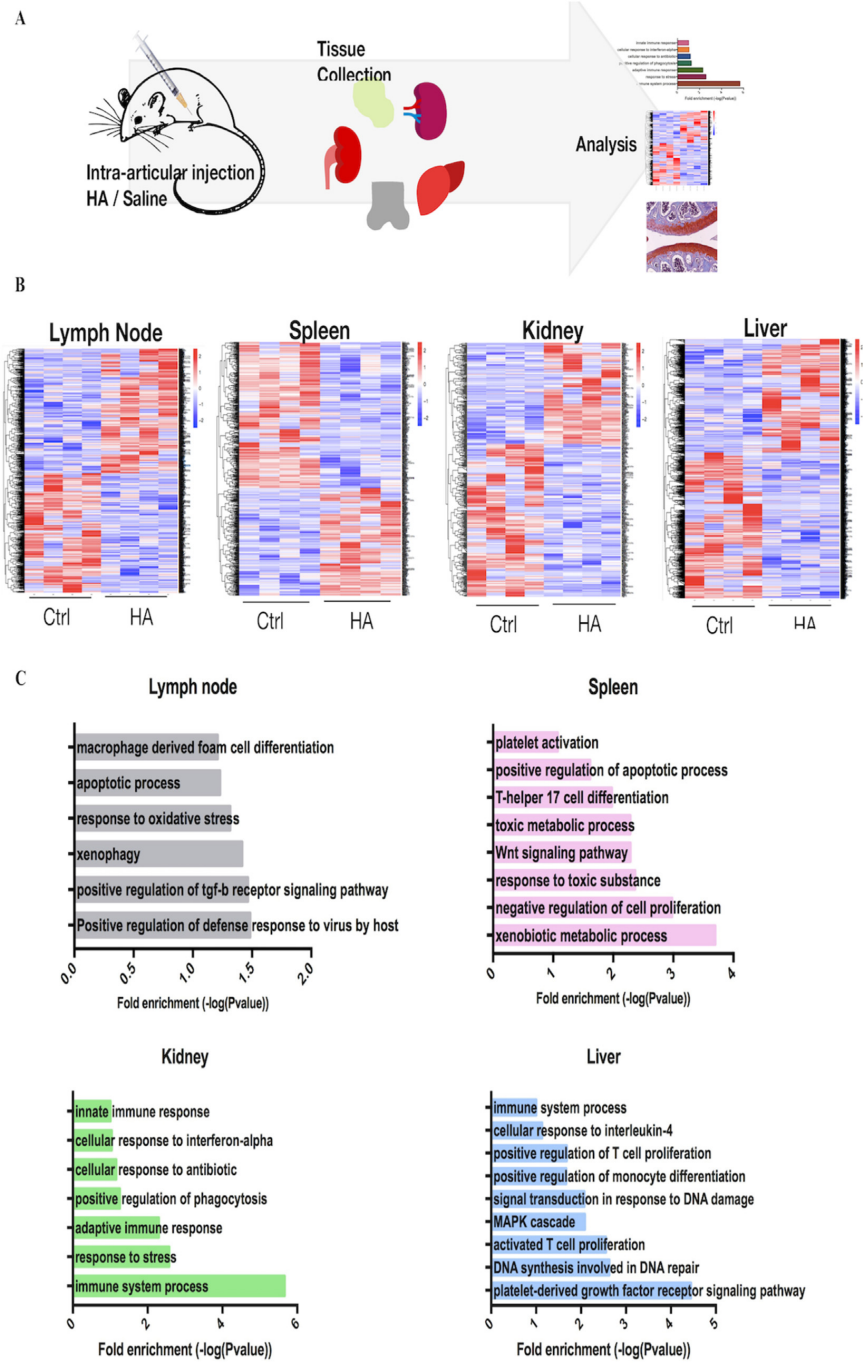
**Systemic influence of intra-articular injected HA on tissue specific gene expression** (A) A flow diagram of the study: lymph node, spleen, kidney, and liver were collected after treatment (B) Heapmaps illustrating DEGs of all four tissues compared with control (C) GO analysis of the relevant upregulated genes in different organs in intra-articular HA-injected mice.

Since HA was illustrated to have an effect on the local area (knee joints and chondrocytes), we also assessed the effect of articular-injected HA in different tissues to evaluate the body's systemic responses as shown in Fig. 3A. Analysis of DEGs of the various tissues from the two groups illustrated that HA injection significantly alters all the transcriptomic profiles of the four selected tissues (Fig. 3B, S4). GO enrichment analysis showed that intra-articular injection of HA elicited pro-inflammatory innate immune response in all organs. The lymph node illustrated GO terms associated with apoptotic process and macrophage derived foam cell differentiation, while the resulted spleen's GO terms were related to xenobiotic metabolic process, positive regulation of apoptosis, and T-helper 17 ​cell differentiation (Fig. 3C). The kidney expressed GO terms linked with immune system process, and adaptive and innate immune responses; similarly, GO terms associated with T cell proliferation, positive regulation of monocyte differentiation and T cell proliferation, and immune system process were observed in the liver (Fig. 3C). GO terms correlated with pathways were also illustrated as the lymph node, spleen and liver expressed positive regulation of TGF-β receptor, Wnt, and platelet-derived growth factor receptor (PDGF) signaling pathway, respectively. TGF-β receptor and Wnt signaling pathways are critical regulators of T and B cells, and PDGF signaling pathway has been reported to serve crucial roles in the development and prognosis of hepatic fibrosis [[49], [50], [51]].

**Fig. 4:**
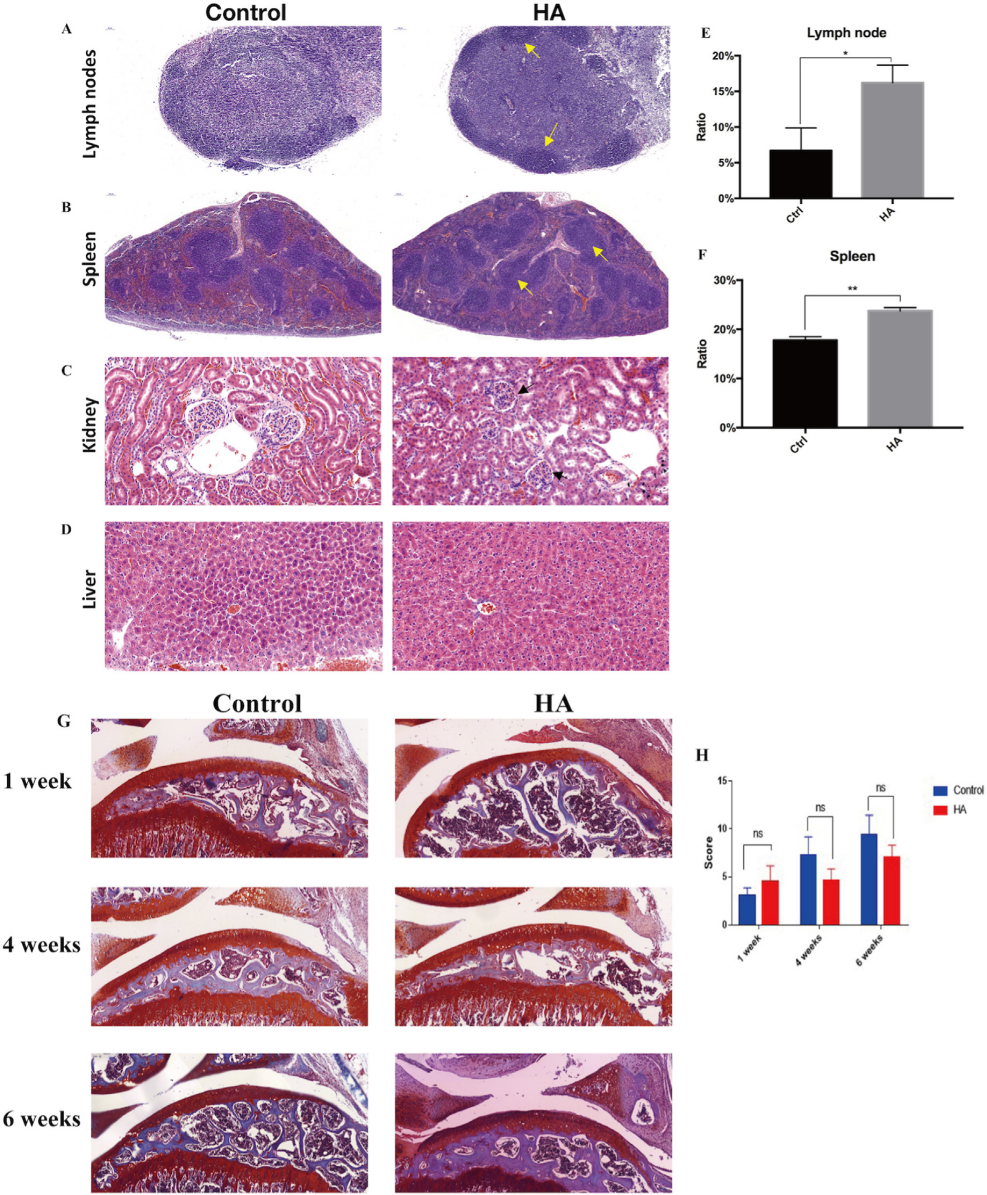
**Histological evaluation of post-HA injection and OA model,** including the (A) lymph node (magnification x20; scale bar: 50um), (B) spleen (magnification x10; scale bar: 100um), (C) kidney (magnification x40; scale bar: 50um), (D) and liver (magnification x40; scale bar: 50um). Quantitative evaluations of the white pulp and germinal center areas in (E) lymph node and (F) spleen (ratio%), (G) Histological sections of the HA-injected knee joints in OA model. Original magnification x20; scale bar: 50um, (H) ICRS scoring on cartilage.

Tissues were also collected for histological analysis. In the lymph node, lymphatic follicles in the outer cortex, mostly containing B cells, appeared to be more intense when compared to the control group; germinal centers, the lighter staining area within the follicle, were also observed in the HA-injected group, indicating B-cell proliferation (Fig. 4A,E) [30]. Similarly, the spleen is another crucial secondary lymphoid organ; HE staining results illustrated that red and white pulps occupied a higher area in the spleen of HA-injected mice (Fig. 4B,F). The white pulp of the spleen also consists of germinal centers of rapidly proliferating B cells surrounded by T cells, macrophages, and dendritic cells. Histological staining images of the kidney also showed abnormal glomerulus and Bowman's capsule; however, no obvious differences were observed in the liver (Fig. 4C and D). Thus, these outcomes further confirmed the *in vitro* results, indicating that HA injections induced inflammatory responses both locally and systemically. Thus, these outcomes further confirmed the *in vitro* results, indicating that HA injections induced inflammatory responses both locally and systematically.

### 3.4 Impact of HA on OA animal model

HA was injected into OA mouse knee joints and the control group was injected with PBS. No significant difference was observed in articular surface, the thickness of the articular cartilage loss of Safranin-O/fast green staining and the mean ICRS score at all timepoints (Fig. 4G and H).

## 4 Discussion

Clinical practice guidelines are important reference evidence for clinical decision-making. However, the current available guidelines present diametrically opposite conclusions for the administration of HA for knee OA [[2], [3], [4], [5]]. Our analysis pooling the high quality trials indicated that intra-articular injections of HA are not effective, in accordance with the conclusion of the 2015 meta-analysis [6], but more intuitive observation from statistical difference level rather than clinically important improvement. Additionally, the evidence of adverse events indicated that HA may be associated with a greater risk of inflammation reactions. These phenomena were confirmed with transcriptomic data revealing DEGs related to responses to inflammation in the knee joint, with an upregulation of *Mmp13* and pro-inflammatory cytokines IL-1β and TNFα in chondrocytes. Moreover, our group has previously developed a novel strategy to study the BOT response of materials in multiple organs, revealing that the injected biomaterials could differentially influence gene expressions [52]. Similar in our study, multi-organ transcriptomic analysis was performed, where results suggested that the injected HA has an influence at the systemic level; DEGs were observed in the lymph node, spleen, kidney, and liver, with GO terms relating to immune cell activation and responses.

In this study, a novel strategy of integrate meta-analysis and multi-organ transcriptomics was utilized. Meta-analytic approach is a large-scale effort to collect data across different studies to form a generalizable system and make evidence-based decisions [53]. However, Meta-analysis is inherently statistical, merely answering the question, ‘Is it effective?’. To explain “Why was it effective or not effective, basic research is needed; thus, we applied BOTs in order to analyze the data and answer this question. It was interesting that HA will introduce inflammation in chondrocytes which cultured on HA-coated plates and immune response in mouse knee joints; and we also found that DEGs were observed in the lymph node, spleen, kidney, and liver with GO terms relating to immune cell activation and responses in the articular-injected HA group. These phenomena were in accordance with the results of meta-analysis, as we synthesized inflammation-related adverse events outcomes related to knee pain, swelling, effusion, arthralgia. But how they can affect the condition, particularly for OA and the symptoms of OA, the mechanisms need more research to explore.

Although the results of meta-analysis suggested that HA may be associated with a greater risk of adverse events related inflammation, there hasn't been much information for subsequent inflammatory responses due to HA in OA patients. It would be better to found in human whether this response would be transient or sustained, and to confirm our conclusion.

Advancement in computational technologies provides the ability to analyze massive datasets, not restricting to one dataset; a review paper published in *Nature Medicine* by Esteva et al. also discussed the different possibilities in using machine powers to evaluate highly complex functions, from internet databases to genome sequencing [54]. This idea was executed in this study and we believe that rapid gains in scientific progress stand to be achieved when these methods are combinatively implemented throughout scientific and clinical researches.

Nevertheless, some aspects in this study could be improved; first, although this meta-analysis pooled the highest-quality trials, the dosages, formulations, and timing of injections of HA and the severity of OA patients were not uniform and varied across trials. Second, the experimental period in the mouse model after HA-injection was at 1 week, this limiting our knowledge on the possible long-term effects of HA *in vivo.* Additional staining should also be applied in different organs to target relevant organ-specific markers on histological samples. Moreover, although RNA-seq data illustrated supportive conclusions to *in vitro* and *in vivo* results, tests will be performed further to validate the signaling pathways involved as multiple players in inflammatory signaling have been identified.

In summary, this study illustrated that the administered HA influenced gene expression and ontology as well as morphologies in different tissues, activating both systemic and local pro-inflammatory immune responses, possibly limiting its efficacy. Therefore, additional research should be conducted on HA, or on alternative materials, to enhance the therapeutic ability in treating OA. This novel unique strategy proposed in this study can be utilized and adapted for future meta-analysis related research, as progress will most like rely on the complete understanding of previous clinical studies, and computational models for high-resolution screening to elucidate the underlying mechanisms.

## 5 Contributors

Kun Zhao, Ya Wen and Varitsara Bunpetch are co-first authors, contributing equally to the manuscript. Zhao was responsible for the design and study involving meta-analysis, data collection, analysis and interpretation, and writing. Bunpetch and Wen were responsible for the study design, experimental studies, data collection, data analysis, data interpretation. Lin contributed to the RNA seq data analysis, and Hu and Xie participated in animal related experiments. Zhang X. contributed to experimental studies involving RNA seq. Professor Hongwei and Zhang S. provided conceptual ideas and designs and supervised all aspects of this work.

## Funding

The 10.13039/501100012166National Key Research and Development Program of China and 10.13039/501100001809National Natural Science Foundation of China.

## Declaration of interests

We declare no competing interests.
