# Supplementary material for: Hype or hope of hyaluronic acid for osteoarthritis: Integrated clinical evidence synthesis with multi-organ transcriptomics
Source: J Orthop Translat. 2022 Jan 15;32:91–100. doi: 10.1016/j.jot.2021.11.006 (PMC8777245; doi:10.1016/j.jot.2021.11.006)
Supplement: Supplementary file 1 [file mmc1.docx]

**Supplementary Materials**

**Table S1. Search strategy**

| **Database** | **#** | **Search strategy** |
| --- | --- | --- |
| PubMed | 1 | “Osteoarthritis”[Mesh] |
|  | 2 | “degenerative arthritis”[Title/Abstract] OR osteoarthr*[Title/Abstract] OR arthrosis[Title/Abstract] |
|  | 3 | #1 OR #2 |
|  | 4 | “Hyaluronic Acid"[Mesh] |
|  | 5 | “Hyaluronic Acid"[ Title/Abstract] OR Hyalgan[Title/Abstract] OR Durolane[Title/Abstract] OR Synvisc[Title/Abstract] OR  Artzal[Title/Abstract] OR Suplasyn[Title/Abstract] OR  BioHy[Title/Abstract] OR Orthovisc[Title/Abstract] OR  hyaluronan[Title/Abstract] OR hyaluronate[Title/Abstract] OR  viscosupplementation[Title/Abstract] |
|  | 6 | #4 OR #5 |
|  | 7 | (randomized controlled trial[Publication Type] OR controlled clinical trial[Publication Type] OR randomized[Title/Abstract] OR placebo[Title/Abstract] OR clinical trials as topic[Mesh:NoExp] OR randomly[Title/Abstract] OR trial[Title]) NOT (animals [Mesh] NOT (humans[Mesh] AND animals[Mesh])) |
|  | 8 | #3 AND #6 AND #7 |
|  | 1 | MeSH descriptor: [Osteoarthritis] explode all trees |
| Cochrane library | 2 | "degenerative arthritis":ti,ab,kw OR osteoarthr*:ti,ab,kw OR arthrosis:ti,ab,kw |
|  | 3 | #1 OR #2 |
|  | 4 | MeSH descriptor: [Hyaluronic Acid] explode all trees |
|  | 5 | ("Hyaluronic Acid"):ti,ab,kw OR (Hyalgan):ti,ab,kw OR (Durolane):ti,ab,kw OR (Synvisc):ti,ab,kw OR (Artzal):ti,ab,kw OR (Suplasyn):ti,ab,kw OR (BioHy):ti,ab,kw OR (Orthovisc):ti,ab,kw OR (hyaluronan):ti,ab,kw OR (hyaluronate):ti,ab,kw OR (viscosupplementation):ti,ab,kw |
|  | 6 | #4 OR #5 |
|  | 7 | #3 AND #6 |
| EMBASE | 1 | 'osteoarthritis'/exp |
|  | 2 | 'osteoarthr*':ab,ti OR (('degenerative' NEAR/2 'arthritis'):ab,ti) OR 'arthrosis':ab,ti |
|  | 3 | #1 OR #2 |
|  | 4 | 'hyaluronic acid'/exp |
|  | 5 | 'hyaluronic acid':ab,ti OR 'hyalgan':ab,ti OR 'durolane':ab,ti OR 'synvisc':ab,ti OR 'artzal':ab,ti OR 'suplasyn':ab,ti OR 'biohy':ab,ti OR 'orthovisc':ab,ti OR 'hyaluronan':ab,ti OR 'hyaluronate':ab,ti OR 'viscosupplementation':ab,ti |
|  | 6 | #4 OR #5 |
|  | 7 | 'crossover procedure':de OR 'double-blind procedure':de OR 'randomized controlled trial':de OR 'single-blind procedure':de OR random*:de,ab,ti OR factorial*:de,ab,ti OR crossover*:de,ab,ti OR ((cross NEXT/1 over*):de,ab,ti) OR placebo*:de,ab,ti OR ((doubl* NEAR/1 blind*):de,ab,ti) OR ((singl* NEAR/1 blind*):de,ab,ti) OR assign*:de,ab,ti OR allocat*:de,ab,ti OR volunteer*:de,ab,ti |
|  | 8 | #3 AND #6 AND #7 |
|  | 9 | #8 AND [embase]/lim NOT ([embase]/lim AND [medline]/lim) |


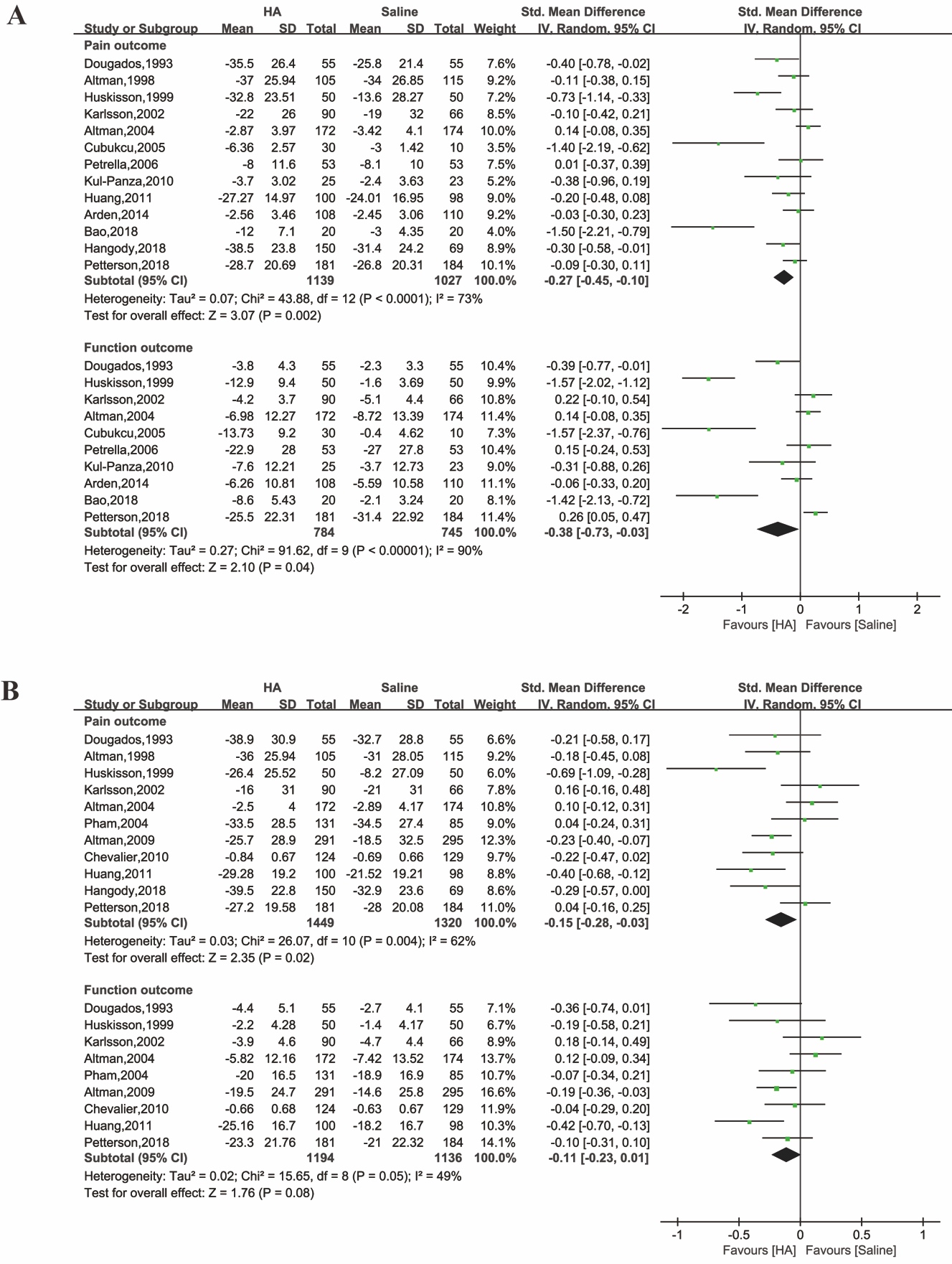
**Figure S1.** The effect of HA compared with saline (A) at short-term and B. at long-term measurement points.

**Figure S2.** Evaluation of chondrocyte proliferation when cultured in different HA concentrations.
